# Reviewing The Benefits of Health Workforce Stability

**DOI:** 10.1186/1478-4491-8-29

**Published:** 2010-12-14

**Authors:** James Buchan

**Affiliations:** 1 Professor, Queen Margaret University, Edinburgh, UK

## Abstract

This paper examines the issue of workforce stability and turnover in the context of policy attempts to improve retention of health workers. The paper argues that there are significant benefits to supporting policy makers and managers to develop a broader perspective of workforce stability and methods of monitoring it. The objective of the paper is to contribute to developing a better understanding of workforce stability as a major aspect of the overall policy goal of improved retention of health workers. The paper examines some of the limited research on the complex interaction between staff turnover and organisational performance or quality of care in the health sector, provides details and examples of the measurement of staff turnover and stability, and illustrates an approach to costing staff turnover. The paper concludes by advocating that these types of assessment can be valuable to managers and policy makers as they examine which policies may be effective in improving stability and retention, by reducing turnover. They can also be used as part of advocacy for the use of new retention measures. The very action of setting up a local working group to assess the costs of turnover can in itself give managers and staff a greater insight into the negative impacts of turnover, and can encourage them to work together to identify and implement stability measures.

## Introduction

This paper examines the issue of workforce stability and turnover in the context of policy attempts to improve retention of health workers. Staff turnover is often the primary topic for monitoring and research when retention is being examined, and can give insights into trends in outflow from the health care organisation. This is particularly relevant at a time of global HRH shortages [1,2]. However a focus only on turnover- on those who leave, is only part of the picture. Policy makers and managers also require an insight into why some staff stay, and what "staying" and workforce stability can contribute to, service delivery, staff workload, and the multiple dimensions of organisational performance, including costs. This paper argues that there are significant benefits to supporting policy makers and managers to develop a broader perspective of workforce stability and methods of monitoring it. The objective of the paper is therefore to contribute to developing a better understanding of workforce stability as a major aspect of the overall policy goal of improved retention of health workers.

## Background

Why should the issue of health workforce stability be important? Retaining and developing the workforce ("talent management") is generally regarded as a major human resource objective for any organisation. In health care there is a general assumption that staff turnover (the opposite of stability), will negatively effect both access to care, and the level and quality of healthcare being provided. Turnover may reduce staffing and patient contact time; can add to organisational costs, if temporary cover for staff who leave (e.g. overtime pay) and recruitment of replacements incurs additional costs; and may reduce individual and organisational performance through the loss of experienced staff, and by undermining teamwork [3,4].

## Methods

The paper is based on a desk review of published and official sources, and analysis of workforce data from official sources.

## Results and Discussion

There is a paucity of research which fully examines the complex interaction between staff turnover and organisational performance, especially quality of care in the health sector. There have been some exploratory studies, and some which have looked at staffing levels and outcomes, and examined turnover rates as one proxy measure for variations in staff satisfaction [5-9]. Few have taken the impact of turnover or stability rates on performance as the primary focus of examination.

Where such research has been conducted, mainly in developed countries, and focusing on nursing staff, there are some powerful messages for policy makers and managers. For example, one US study reported that health care organizations with the lowest nurse turnover rates (less than 12 percent) had the lowest risk-adjusted mortality scores, as well as the lowest severity-adjusted length-of-stay, and that for health care organizations with turnover rates in excess of 22 percent, the severity adjusted average length-of-stay was 1.2 days longer than those with the lowest turnover rates. The authors noted that whilst these findings do not establish a causal relationship, they did suggest that higher rates of turnover among the nursing staff probably lead to decreased efficiency and productivity, which in turn affects patient care [10]. Other detailed research studies are underway which will focus on the links between turnover and measures of outcome [11,12]; others have also identified the need to examine the impact of so-called staff "churn": a continuous high level of turnover, often accompanied by vacancies and reliance on short term cover by temporary staffing[13].

Whilst there may be relatively little research evidence on the impact of health workforce turnover/stability on care outcomes, there remains a need for organisations to be able to measure workforce turnover and stability, and assess its impact on costs..

Most research which focuses on health worker retention and which attempts to measure it uses turnover as a main indicator. Turnover, and the alternate terms of "attrition" or "wastage" are usually expressed in terms of the % of staff of a particular occupation or workplace who have left the organisation (or have moved jobs) within the last twelve months. The terms "attrition" and "wastage" are normally applied when the measurement is the % of staff who have left the organisation or system under scrutiny, whilst "turnover" is more often applied if all job moves (including those within the system) are being assessed [1-3,14].

This type of turnover data is routinely used as a method of comparing the "leaving" rates in different workplace units or organisations, and can be a method of benchmarking variations in rates across systems or organisations. A number of standard measures of turnover can be used, the main requirement being consistency of definition and application, to allow comparison over time and between employing units. The recent WHO/World Bank/USAID handbook [2] has suggested that the "workforce loss ratio" can be calculated by using number of workers who have left in the last year as numerator, and total number of health workers as the denominator^. ^This is the standard approach- determining the loss over the year as a proportion of the total workforce [see also [14]].

If the reason for monitoring is to assess the ability of the organisation or system under scrutiny to retain staff it is important that any monitoring can differentiate between permanent and temporary moves, and what has been termed "involuntary" turnover or attrition (e.g. statutory retirement, ill health, death) and "voluntary" turnover, resignation or attrition [2,15]. Research using turnover or attrition data can give policy makers an insight into varying rates in different cadres of worker, and different reasons for attrition. One recent study, for example, highlighted that attrition of doctors and registered nurses in Kenya was much higher at provincial hospitals than at district hospitals or health centres, whereas the opposite pattern was found for laboratory staff and pharmacists [16]. It also found that resignation was the main factor in attrition of doctors and clinical officers, whilst the main reason for attrition of nurses was retirement.

One limitation of the standard measure of turnover (leavers in the year/total workforce) is that over the time period under measure it does necessarily differentiate well when comparing units with high and repeated turnover in a few posts, and those with lower turnover in more posts [13,17]. This can limit its utility as a measure of retention as it measures the leaving rate and gives less insight into how many staff are staying, and for how long. As noted earlier, a focus on turnover is a focus on the "leavers", and there should also be some consideration of "stayers" if retention is the focus. Developing an understanding of the stability in the workforce can provide managers and policy makers with a better understanding of the labour dynamics both internally, within the organisation, and externally, in terms of how the organisation connects to the wider labour markets. There are several different possible measures of workforce stability which can be used to give an insight into how many staff stay with the organisation, for what time period.

One indicator which can be used to assess workforce stability is the average years in post reported by staff group or work location [18]. A second measure of stability is to calculate the stability rate or "index" for each location, occupational group or profession. The stability index assesses the proportion of staff who were in post at the beginning of the year who were still in post at the end of the year. As data is collected over a longer period of time, stability indices for 1 year, 2 years (i.e the % of staff who have remained in post at end of two years), and longer can be calculated, giving a better insight into the relative rates of retention of different types of staff, or different organisations within the system under scrutiny. This type of measure may be of utility in most healthcare settings where HRH data collection is limited, rather than more sophisticated indicators that require

Table 1 gives an example of the use of stability index data. It is based on data collected by the National Health Service (NHS) in Scotland. There are 14 main regional employing organisations (Boards) within the NHS in Scotland and the Table presents the one and two year stability rates for all staff nationally, and in each of the fourteen Boards. The overall workforce (full time equivalent) is approximately 130,000, including approximately 11,000 doctors and 40,000 registered nurses. Boards vary significantly in size; the largest employs over 35,000 staff. Two year stability rates for nurses and doctors are also shown. The rates are calculated by estimating the percentage of staff that were in substantive posts at 30 September and who were still in substantive posts within the National Health Service (NHS) in Scotland in the same NHS Board and the same staff group a year later (index 1), and two years later (index 2).

**Table 1:** 2 year stability rate (%), NHS Scotland, selected staff groups and all staff, by regional Boards (A to N).

	Nursing	Medical	All staff	*All staff 2007-8*
All	85.1	82.8	75.4	*88.4*
A	89.3	84.5	67.0	*84.8*
B	85.5	80.0	73.0	*86.0*
C	85.3	80.6	73.8	*87.0*
D	85.0	83.7	74.2	*88.4*
E	85.4	83.7	74.9	*89.1*
F	88.9	83.5	76.4	*89.5*
G	82.0	87.2	77.3	*89.7*
H	82.3	81.8	77.7	*90.3*
I	81.2	86.7	77.7	*90.5*
J	87.0	83.1	78.0	*90.6*
K	87.0	87.7	78.6	*91.0*
L	88.0	92.6	79.6	*91.1*
M	88.3	76.8	80.0	*91.3*
N	81.3	72.2	80.5	*92.6*

(1 year stability rate for all staff in italics)

Source: Scottish Workforce Information Standard System (SWISS)

The data is presented for each Board, in anonymised form, with a ranking of the two year stability rate for all staff (lowest stability rate at top- "Board A"). A quick examination of the data in Table 1 provides some immediate insights and underlines how useful stability data can be, particularly if it is collated beyond one year. For example it is evident that there is marked variation between Boards in the stability rate for all staff - the 2 year rate varies between 67% and 80.5%. The two year stability rates for doctors and for nurses are higher than the average for all staff groups. This is perhaps not unsurprising as these health professional groups are better paid and more likely to be located within career structures which will assist in retaining them in the health sector than are some "support" staff in clerical and administrative jobs; furthermore, as health professionals their skills will be less transferable to non health settings. There is also variation in the ranking of the two year rate- Board A which has the lowest stability rate for all staff, does not report the lowest rate for doctors (Board I) or nurses (Board N). Assessment of the data by size of Board can also give some indication of the possible impact of organisation size, but also has to be qualified by the recognition that "smaller" Boards (by workforce size) tend to be relatively rural, remote or island based.

This relatively straightforward measure of stability can assist in inform managers and policy makers about which occupations have relatively good or poor stability, or which organisations are retaining proportionately more staff for more time. As such it can point to "problem" areas. These may be areas with low stability where greater staff stability is required. The analysis can also identify organisations with high desired stability rates which may have best practice methods that can be identified and networked.

One of the benefits of this type of measure of stability is its utility. With some caveats about interpretation of data where the workforce populations are small [13,17] the stability rate is simple to measure, and simple to understand. It can be no more complex to collect data on stability than it is on turnover. Where new HR information systems are being developed, or current systems upgraded, consideration should be given to collating and standardising the use of the stability rate indicator. More complex measures of stability can also be used if an employing organisation wishes to develop a more detailed insight into stability and the length-of-service structure of the staff to facilitate inter-organisation comparisons [19].

Whilst routine collection and analysis of turnover and stability data can give an insight into level of staff retention and reasons for staying, or leaving, an estimate of the costs of turnover can assist in raising awareness of the need to improve retention, and can contribute to advocacy for policy interventions to improve retention [3,8,10,20,21]. In addressing issues related to costs of turnover, it is important first to note that there can benefits as well as costs to the employing organisation when it experiences staff turnover (see Table 2). The critical issue, as noted earlier, is that any unplanned and unnecessary ("voluntary") turnover should be prevented, if at all possible, in the interests of achieving greater stability in the workforce.

**Table 2:** Potential Organisational Costs and Benefits of Staff Turnover

Costs	Benefits
Loss of Experienced staff	'New Blood'
- Lost Knowledge	- New Knowledge
- Decreased Morale	- Improve Morale
Constraint on Quality/Level of Service	Allows Career Progression
Separation Costs	Increase in Organisation Flexibility: e.g. skill mix change
Temporary Replacement Costs	Opportunity for Cost Reduction/Consolidation
Recruitment Costs	Decrease in other 'Withdrawal' Behaviour eg Absenteeism
Induction/Training Costs	

Source: adapted from (3)

From a management perspective, the potential benefits of some level of turnover can include 'freeing up' of posts to allow new staff with new ideas and energy into the organisation, opportunities for cost reduction through staff reduction or deployment, and through 'losing' disaffected staff who would otherwise exhibit other forms of withdrawal behaviour, such as absence from work. There is however no consensus on what is the "ideal" level of turnover[14], and some potential benefits may be only short term.

However, as noted earlier in the paper it is clear that there can be a range of organisational costs and negative impacts on care associated with any unnecessary turnover of staff. Assessing the cost of turnover should therefore be an integral element in the approach to maintaining workforce stability. If the typical or average cost of turnover of staff is known, this can assist in assessing the impact on the organisation of different levels of turnover, and can also provide managers with an indication of the cost effectiveness of improving retention through turnover reduction (or stability increase) strategies.

There is little published data or information on turnover costs in the health sector and much of that which does exist stems from the United States. Most studies attempt to arrive at a cost per individual staff member "leaving", and then calculate a total organisational cost per annum. Costs per staff are usually examined in at least four components- separation costs (the costs incurred by the staff member leaving), temporary replacement costs (the costs of covering the post made vacant by the staff member leaving- e.g use of an agency staff, sue of overtime work by remaining staff etc), recruitment costs (the costs in advertising and selecting the replacement, and providing relocation costs), and induction costs (including "lost" productivity, until the replacement reaches the same level of productivity as the staff member who had left) [3,8,20,21]. Initial costs of training the worker are usually not included.

It should be recognised that actual turnover costs may vary significantly between individual employees, depending on the grade and experience of the worker, and on the replacement strategy used by the employer [3,20,21]. For experienced staff at senior level it is likely that turnover costs will be significantly higher than for junior staff, due to longer periods of induction and, in some specialities and occupations, because of skills shortages and difficulties in recruitment.

Turnover costs will also vary depending upon the replacement strategy being adopted (e.g. replacing an experienced worker with a less experienced worker is likely to lead to lower replacement costs, but lower productivity, in the short term at least) and are likely to vary according to the clinical and geographical setting.

There are a number of ways that the overall impact of staff turnover costs at organisation level can be illustrated, such as:

• percentage of paybill;

• cost per patient day;

• cost saving of reduction in turnover

Table 3 gives an example using % of paybill.. Using assumptions of 7 per cent turnover and turnover costs of $8,000 per nurse in an organisation which employs 500 nurses, this would be equivalent to turnover costs of $280,000 per annum. If the turnover was reduced to 5 per cent per annum, the illustrative example suggests a cost saving to the organisation of $80,000 per annum.

**Table 3:** Illustrative Examples of the use of Turnover Costs Data

a) Nurse Turnover Costs as a percentage of the paybill	b) Cost Saving of reduction in Turnover
Turnover cost = 500 × 7% × $8000 = $280,000	[assumes turnover reduced from 7% to 5%]
Paybill = 500 × $20,000 = $10,000,000	Saving = 500 × (7%- 5%) × $8000 = $80,000
Turnover costs = 2.8% of paybill	

Source: Author

This approach to estimating turnover costs ensures that there is sufficient detail at the level of the individual 'leaver' to allow aggregated data to be used as a 'not less than' total cost to the organisation. The example in Table 3 above illustrates the potential magnitude of turnover costs at organisational level, and also reveals the potential cost savings which management could achieve by reducing turnover. Clearly, as noted above, not all turnover is "bad" for the organisation, but any turnover which could have been prevented through management action would have reduced turnover costs. Improving workforce stability can carry with it a benefit to the organisation in terms of reduced cost.

One recent study in the US [22] estimated that total turnover costs for a hospital system employing 5000 employees was between $US17 and $29 million. Another study [21] focusing on costs of individual turnover rather than organisational, highlighted nurse turnover cost assessments of between US$21,500 and US$31,500 per nurse, with a rough "rule of thumb" that the cost of staff nurse turnover normally fell between 0.75 and 2.0 times annual salary, depending on replacement strategy and other factors.

## Conclusion

Intuition would suggest that improving retention and stability of the health workforce brings benefits to staff, the organisation and those being cared for. The limited research available does provide some support for this, and gives insight into how health workforce stability can contribute to reduced costs, improved productivity and better care outcomes. Of equal importance, and more immediate utility in most organisations, is the possibility of introducing a measure or indicator of staff stability, and of examining the costs of staff turnover. These types of assessment can be valuable to managers and policy makers as they examine which policies may be effective in improving stability and retention, by reducing turnover. They can also be used as part of advocacy for the use of new retention measures. An additional benefit can be that the very action of setting up a local working group to assess the costs of turnover can in itself gives managers and staff a greater insight into the negative impacts of turnover, and can encourage them to work together to identify and implement stability measures.

## Competing interests

The authors declare that they have no competing interests.

## Authors' contributions

JB conceived, researched and wrote the paper
